# The predictive value of preoperative ultrasound contrast parameters for early recurrence after transarterial chemoembolization for hepatocellular carcinoma

**DOI:** 10.3389/fmed.2026.1748609

**Published:** 2026-09-04

**Authors:** Shuqiang Jin, Qimeihui Wang, Lingyu Zhu, Dengke Teng, Guoqing Sui, Hui Wang

**Affiliations:** Department of Ultrasound, China-Japan Union Hospital of Jilin University, Jinlin, China

**Keywords:** contrast-enhanced ultrasound, early recurrence, MTT, PI, transarterial chemoembolization for hepatocellular carcinoma, TTP

## Abstract

**Objective:**

To analyze the predictive value of preoperative contrast-enhanced ultrasound (CEUS) parameters for early recurrence after transarterial chemoembolization (TACE) for hepatocellular carcinoma (HCC).

**Methods:**

A retrospective study was conducted on 242 HCC patients treated at our hospital between May 2021 and May 2023. Based on recurrence within 24 months post-TACE, patients were categorized into an early recurrence group (*N* = 117) and a non-early recurrence group (*N* = 125). We collected baseline data and performed quantitative analysis of CEUS parameters, including time to peak (TTP), mean transit time (mTT), peak intensity (PI), and rise time (RT). A multivariable logistic regression model was used to identify risk factors for early recurrence. The predictive performance of the parameters was assessed using ROC curves, and recurrence rates were compared with Kaplan-Meier analysis.

**Results:**

Significant differences were found between the two groups in alpha-fetoprotein (AFP), carbohydrate antigen 199 (CA199), aspartate aminotransferase (AST), alanine aminotransferase (ALT), gamma-glutamyl transpeptidase (GGT), alkaline phosphatase (ALP), total bilirubin (TBIL), tumor maximum diameter, and rapid washout in the portal phase (all *P* < 0.05). The early recurrence group had significantly lower TTP and mTT, and higher PI compared to the non-early recurrence group (all *P* < 0.001). No significant difference was found in RT (*P* > 0.05). Multivariable logistic regression analysis identified elevated AFP, CA199, GGT, and PI as independent risk factors for early recurrence, while prolonged TTP and mTT were independent protective factors (all *P* < 0.05). Of note, although CA19-9 is traditionally considered a biomarker for intrahepatic cholangiocarcinoma, its elevation in our strictly defined HCC cohort (pathologically confirmed or LI-RADS LR-5) may reflect aggressive tumor biology rather than inadvertent inclusion of non-HCC patients. The combined prediction model (TTP + mTT + PI) had a significantly superior AUC (0.975) for predicting early recurrence compared to any parameter alone (all *P* < 0.001). Internal validation using 1,000 bootstrap resamples yielded a bias-corrected AUC of 0.968 (95% CI: 0.941–0.985), confirming the model’s robustness against overfitting. Patients with low TTP or low mTT had significantly higher recurrence rates, whereas those with low PI had significantly lower recurrence rates (all *P* < 0.001).

**Conclusion:**

The combined detection of the CEUS parameters TTP, mTT, and PI can effectively assist in predicting early recurrence in HCC patients after TACE.

## Introduction

Hepatocellular carcinoma (HCC) is one of the six most common malignant tumors worldwide and the third leading cause of cancer-related deaths, posing a significant challenge to public health systems due to its high incidence and mortality rates (1). China, as a country with a high incidence of liver cancer, accounts for 42.5% of new cases worldwide, with a liver cancer mortality rate of approximately 28.5 per 100,000, representing 15% of all cancer deaths, and a 5-year survival rate of only 12–15%, significantly lower than that of breast cancer, lung cancer, and other tumor types (2–4). Although systemic therapies such as targeted drugs and immunotherapy have made breakthrough progress in recent years, transarterial chemoembolization (TACE) remains the core treatment for liver cancer patients, particularly for unresectable lesions or cases of recurrence and metastasis after surgery (5). However, the high early recurrence rate after TACE has become a key bottleneck limiting long-term survival (6). Therefore, exploring preoperative imaging biomarkers to accurately predict recurrence risk has become an urgent need to optimize individualized treatment plans and improve outcomes.

Contrast-enhanced ultrasound (CEUS) is an emerging imaging technique that uses microbubble contrast agents to enhance tissue blood flow signals, enabling dynamic observation of tumor angiogenesis and microcirculatory characteristics (7, 8). Compared to conventional ultrasound, CEUS demonstrates higher sensitivity and specificity in the qualitative diagnosis of hepatocellular carcinoma, particularly in detecting sub-centimeter lesions smaller than 1 cm (9). Its quantitative analysis parameters, such as time to peak (TTP), mean transit time (mTT), peak intensity (PI), and rise time (RT), objectively reflect the degree of tumor vascularization and hemodynamic status. Previous studies have shown that CEUS features such as irregular vascular morphology and multiple blood supply within tumors are associated with the malignancy of hepatocellular carcinoma and postoperative recurrence (10, 11), but systematic research on its predictive value for early recurrence after TACE remains lacking. Existing imaging prediction models primarily rely on postoperative pathological features or enhanced CT/MRI data, which have limitations such as complex procedures, radiation exposure, or contrast agent deposition interference. CEUS, with its non-invasive, real-time, and reproducible nature, offers unique advantages in the comprehensive management of hepatocellular carcinoma. However, current research has primarily focused on the diagnostic and therapeutic efficacy assessment value of CEUS, with insufficient exploration of its clinical significance in predicting postoperative recurrence after TACE. This study aims to systematically analyze the association between preoperative CEUS quantitative parameters (TTP, mTT, PI, RT) and early recurrence after TACE for hepatocellular carcinoma. It seeks to explore their clinical translational value, providing scientific evidence for preoperative risk stratification and individualized treatment decisions. The study results are expected to fill a gap in this field, promoting the expansion of CEUS from a diagnostic tool to a prognostic predictive biomarker, ultimately contributing to the realization of precision medicine for hepatocellular carcinoma.

## Materials and methods

### Study population

A retrospective selection of 289 patients with liver cancer admitted to our hospital between May 2021 and May 2023 was conducted. Based on inclusion and exclusion criteria, 242 patients were ultimately selected as the study population. All included patients had either histopathologically confirmed HCC with immunohistochemical evidence (Hep Par-1 or glypican-3 positivity, CK7/CK19 negativity) or met LI-RADS LR-5 criteria on preoperative contrast-enhanced CT/MRI. Patients with ICC, cHCC-CCA, or indeterminate LI-RADS categories (LR-3, LR-4, or LR-M) were excluded. Patients were divided into an early recurrence group (*N* = 117) and a non-early recurrence group (*N* = 125) based on liver cancer recurrence within 24 months after TACE. This study was approved by the Ethics Committee of our hospital and complies with the Declaration of Helsinki.

### Inclusion and exclusion criteria

Inclusion criteria: ➀ Pathologically confirmed hepatocellular carcinoma (HCC) meeting the diagnostic criteria outlined in the Chinese Clinical Practice Guidelines for the Diagnosis and Treatment of Hepatocellular Carcinoma (12), with immunohistochemical staining [positive for hepatocyte paraffin antigen-1 (Hep Par-1) or glypican-3, and negative for cytokeratin 7 (CK7) and cytokeratin 19 (CK19)] to exclude intrahepatic cholangiocarcinoma (ICC) and combined hepatocellular-cholangiocarcinoma (cHCC-CCA). For patients without pathological confirmation, a definitive Liver Imaging Reporting and Data System (LI-RADS) category 5 (LR-5) classification on contrast-enhanced CT or MRI was required for inclusion; ➁ Initial treatment modality was transarterial chemoembolization (TACE), with no prior surgical or other anticancer treatments such as radiotherapy or chemotherapy; ➂ Child-Pugh liver function classification grade A or B; ➃ Complete clinical and follow-up data; ➄ No severe abnormalities in cardiac, pulmonary, or renal function; ➅ American Society of Anesthesiologists (ASA) physical status classification of 1–3.

Exclusion Criteria: ➀ Invasive hepatocellular carcinoma or recurrent hepatocellular carcinoma; ➁ Concurrent coagulation or immune dysfunction; ➂ Concurrent other malignant tumors; ➃ Distant metastasis; ➄ Allergy to contrast agents; ➅ Recent acute coronary syndrome, acute heart failure, severe heart failure, or severe pulmonary hypertension, among other serious cardiovascular or respiratory diseases; ➆ Concurrent severe infectious diseases; ➇ History of liver transplantation, etc.

### Medical record data collection

Collect patient age, gender, body mass index (BMI), smoking history, drinking history, hepatitis B, Child-Pugh classification, Barcelona Clinic Liver Cancer (BCLC) staging, alpha-fetoprotein (AFP), carcinoembryonic antigen (CEA), carbohydrate antigen 199 (CA199), aspartate aminotransferase (AST), alanine aminotransferase (ALT), γ-glutamyl transpeptidase (GGT), alkaline phosphatase (ALP), cholinesterase (ChE), albumin (ALB), total bilirubin (TBIL), tumor maximum diameter, histological differentiation grade, CEUS examination findings (tumor margin characteristics, arterial phase hyperenhancement, rapid washout in the portal phase), etc.

### LI-RADS classification and imaging review

All preoperative contrast-enhanced CT and MRI examinations were independently reviewed by two abdominal radiologists (with 8 and 12 years of experience, respectively) who were blinded to clinical outcomes. Each lesion was categorized according to the LI-RADS v2018 criteria. Lesions were classified as LR-5 (definitely HCC) if they met the following criteria: non-rim arterial phase hyperenhancement (APHE) plus washout appearance (non-peripheral, rim, or delayed) and an enhancing capsule. For patients without pathological confirmation, only those with a definitive LR-5 classification were included in the final cohort. Inter-observer agreement for LI-RADS categorization was assessed using Cohen’s kappa coefficient (κ = 0.87, 95% CI: 0.81–0.93), indicating excellent agreement.

### Transarterial chemoembolization (TACE) treatment

Under digital subtraction angiography guidance, the femoral artery is punctured using the Seldinger technique, and the catheter is inserted into the tumor-supplying artery. Take 10 mL of iodized oil injection solution (Shanghai Xudong Haipu Pharmaceutical Co., Ltd.) and add 30 mg of injectable hydrochloride pirarubicin (Zhejiang Hai Zheng Pharmaceutical Co., Ltd.) to form a suspension, which is slowly infused until the entire tumor is filled. Subsequently, 50–100 mg of gelatin sponge particle embolization agent (Hangzhou Ailike Pharmaceutical Technology Co., Ltd.) is used for arterial embolization. Adjust the dosage of the drugs used according to the size and number of tumors. After completing the surgery, perform hemostasis and bandaging at the femoral artery puncture site.

To minimize treatment heterogeneity, all TACE procedures were performed by the same interventional radiology team using a standardized protocol as described above. The dosage of chemotherapeutic agents (pirarubicin: 30 mg fixed dose) and embolization materials (iodized oil: 10 mL fixed volume; gelatin sponge particles: 50–100 mg based on tumor size and vascularity) followed institutional protocols. No patients received concurrent systemic therapy (targeted therapy or immunotherapy) before documented recurrence, ensuring that recurrence events reflected TACE treatment failure rather than confounding by additional treatments.

### Contrast-enhanced ultrasound (CEUS) examination

CEUS examination was performed 1 day prior to TACE. A GELOGIQE9 color Doppler ultrasound diagnostic instrument was used, with a mechanical index of 0.06 and a probe frequency of 2–4 MHz. SonoVue contrast agent (Bracco, Italy) is used. A standard ultrasound examination is first performed, with detailed documentation of tumor number, size, shape, borders, echogenicity, and blood supply. The most suitable cross-section for clearly visualizing the tumor is selected as the quantitative analysis cross-section. The ultrasound probe is stabilized, and the patient is instructed to maintain steady breathing. The system is switched to CEUS mode. SonoVue contrast agent (2.4 mL) is injected via the superficial veins of the forearm, followed by 5 mL of 0.9% sodium chloride solution. Timing begins simultaneously, and dynamic images of the entire contrast process are observed and saved for subsequent data analysis. The enhanced phases include the arterial phase (0–30 s), portal venous phase (31–120 s), and delayed phase (121–300 s). After data acquisition, the dynamic CEUS image data were processed using the SonoLiver quantitative analysis software (TomTec, Germany) to extract quantitative parameters, including time to peak (TTP), mean transit time (mTT), peak intensity (PI), and rise time (RT).

Regarding region of interest (ROI) selection for quantitative analysis, a circular ROI of 5–8 mm in diameter was manually placed within the most highly vascularized area of the target lesion (the area with the strongest contrast enhancement during the arterial phase), avoiding necrotic, cystic, or hemorrhagic regions as identified on B-mode ultrasound and CEUS images. The ROI was positioned at least 2 mm away from the tumor margin to minimize partial volume effects and contamination from adjacent liver parenchyma. For each target lesion, three consecutive measurements were performed, and the average values were used for final analysis. In patients with multiple lesions (≤ 3 lesions), the largest lesion was selected as the target lesion for quantitative analysis. The rationale for selecting the most highly vascularized area within the target lesion is that this region best reflects the active angiogenic activity and aggressive biological behavior of HCC, which are key drivers of early recurrence after TACE. This standardized ROI placement protocol was performed by two experienced radiologists (with > 5 years of experience in CEUS) who were blinded to the patients’ clinical outcomes, and inter-observer agreement was assessed using the intraclass correlation coefficient (ICC > 0.85 for all parameters).

### Recurrence assessment and endpoint definition

Postoperative follow-up patients undergo serum alpha-fetoprotein level testing, liver function tests, and enhanced CT or MRI screening of the chest and abdomen every 3 months to screen for HCC recurrence (13). The primary endpoint was early recurrence, defined as intrahepatic or extrahepatic recurrence detected by imaging (contrast-enhanced CT or MRI) within 24 months after TACE, confirmed by at least two radiologists blinded to the study. The exact time to recurrence (in months) was recorded as the interval from TACE procedure to the date of imaging confirmation of recurrence. For patients without recurrence, follow-up time was censored at the last imaging examination or at 24 months post-TACE, whichever occurred first. Patients who died without documented recurrence or were lost to follow-up before 24 months were censored at the time of death or last follow-up. Kaplan–Meier survival analysis was therefore appropriate as time-to-recurrence data were available for all patients.

### Sample size calculation

Based on a pilot study of 50 HCC patients treated with TACE (20 with early recurrence, 30 without), the expected AUC for the combined model was estimated at 0.85. To achieve 80% power with a two-sided alpha level of 0.05 and an expected AUC of 0.85 under the alternative hypothesis vs. 0.5 under the null hypothesis, a minimum of 98 patients (49 per group) was required using the method of Hanley and McNeil. To account for potential missing data and multiple predictor variables (up to 10 candidate variables), we aimed to recruit at least 200 patients. The final sample of 242 patients (117 with early recurrence, 125 without) exceeded this requirement.

### Statistical analysis

Data statistical analysis and graphing were performed using SPSS 27.0 statistical software (SPSS, Inc., Chicago, IL, United States) and GraphPad Prism 9.5.0 software (GraphPad Software Inc., San Diego, CA, United States) for data statistical analysis and graphing. The Kolmogorov-Smirnov test was used to assess normal distribution. For normally distributed continuous variables, data were expressed as mean ± standard deviation, and comparisons between groups were performed using the independent samples *t*-test. For non-normally distributed continuous variables, data were expressed as median (interquartile range), and comparisons between groups were performed using the Mann-Whitney U test; Categorical data were expressed as counts and percentages, and comparisons between groups were performed using the chi-square test. A multivariable logistic regression model was established to analyze the risk factors for early recurrence after TACE in hepatocellular carcinoma patients, with early recurrence status (0 = no recurrence, 1 = recurrence) as the dependent variable. Odds ratios (ORs) with 95% confidence intervals (CIs) were calculated. Prior to multivariate Cox regression, multicollinearity among continuous independent variables was assessed using variance inflation factor (VIF), with a VIF threshold of > 5 indicating significant collinearity. All VIF values were below 2.5, indicating no significant multicollinearity. Variable selection for multivariate Cox regression was performed using a stepwise forward likelihood ratio method with entry criterion of *P* < 0.05 and removal criterion of *P* > 0.10. ROC curves were plotted to analyze the predictive value of CEUS parameters for early recurrence after TACE in hepatocellular carcinoma patients, and the cutoff values and area under the ROC curve (AUC) were obtained. To assess potential overfitting of the combined model, rigorous internal validation was performed using 1,000 bootstrap resamples with replacement, calculating the bias-corrected AUC and 95% confidence intervals. The optimism-corrected AUC was derived by subtracting the average optimism (the difference between the apparent AUC and the bootstrap AUC) from the apparent AUC. Additionally, 10-fold cross-validation was performed as a complementary internal validation method, where the dataset was randomly partitioned into 10 equal subsets, with 9 subsets used for training and 1 subset for validation, repeated 10 times to obtain the cross-validated AUC. Calibration of the combined model was assessed using the Hosmer–Lemeshow goodness-of-fit test and calibration plots. The robustness of the model was further evaluated by sensitivity analyses using alternative cut-off values and by assessing the stability of the Cox regression coefficients across bootstrap samples. Kaplan-Meier curves were plotted to analyze recurrence-free survival (RFS) after TACE, with the log-rank test used to compare RFS distributions between groups stratified by TTP (≤ 25 s vs. > 25 s), mTT (≤ 43 s vs. > 43 s), and PI (≤ 40 dB vs. > 40 dB). Number-at-risk tables were provided below each Kaplan-Meier curve to facilitate interpretation of censoring patterns. The median recurrence-free survival time was calculated for each group when applicable. *P-*values were two-sided, and *P* < 0.05 was considered statistically significant.

For all logistic regression analyses, continuous variables (AFP, CA199, GGT, TTP, mTT, PI) were entered as continuous measures without dichotomization. The coding direction for PI was such that higher values indicated greater peak contrast intensity. An OR > 1 for PI indicates that increased peak intensity is associated with higher risk of early recurrence, consistent with the observation that the high-PI group (PI > 40 dB) had a recurrence rate of 73.61% compared to 11.22% in the low-PI group (PI ≤ 40 dB).

### Data quality and missing data handling

Of the 289 initially screened patients, 47 were excluded based on prespecified criteria as described above. Among the 242 included patients, no missing data were present for demographic variables, CEUS parameters, or primary laboratory indicators (AFP, CA199, GGT, AST, ALT, ALP, TBIL). For secondary variables, missing data rates were as follows: histological differentiation (0%, complete), tumor margin assessment (0%, complete), and rapid washout in portal phase (0%, complete). Therefore, complete-case analysis was performed without imputation, as the absence of missing data in key analytic variables precluded the need for multiple imputation or other missing data handling techniques.

## Results

### Baseline data comparison

A retrospective review was conducted of 289 patients with hepatocellular carcinoma admitted to our hospital between May 2021 and May 2023. Based on inclusion and exclusion criteria, 242 patients were ultimately selected as study subjects. Patients were divided into an early recurrence group (*N* = 117) and a non-early recurrence group (*N* = 125) based on hepatocellular carcinoma recurrence within 24 months post-TACE. There were no significant differences between the two groups in terms of age, gender, BMI, smoking history, drinking history, hepatitis B, BCLC staging, Child-Pugh classification, CEA, ChE, ALB levels, histological differentiation, and CEUS examination findings (tumor margin status, arterial phase hyperenhancement) (all *P* > 0.05); However, there were statistically significant differences between the two groups in terms of AFP, CA199, AST, ALT, GGT, ALP, TBIL levels, tumor maximum diameter, and rapid washout in the portal phase (all *P* < 0.05), as shown in Table 1.

**TABLE 1:** Comparison of clinical baseline characteristics.

Characteristic	Non-early recurrence (*N* = 125)	Early recurrence (*N* = 117)	Test statistic *(t/*χ*^2^)*	*P*-value
Age (years)	57.14 ± 5.32	56.94 ± 6.03	0.279	0.780
BMI (kg/m^2)^	22.88 ± 2.01	23.01 ± 1.86	0.534	0.594
Gender (m/f, N)	85/40	79/38	0.006	0.937
Smoking history (N,%)	37 (29.60)	39 (33.33)	0.391	0.532
Drinking history (N,%)	45 (36.00)	44 (37.61)	0.067	0.796
Hepatitis B (N,%)	102 (81.60)	97 (82.91)	0.071	0.791
BCLC staging (N,%)	–	–		
Phase B	98 (78.40)	89 (76.07)	0.187	0.665
Phase C	27 (21.60)	28 (23.93)		
Child-Pugh classification (N,%)	–	–		
Grade A	64 (51.20)	62 (52.99)	0.078	0.780
Grade B	61 (48.80)	55 (47.01)		
AFP (ng/mL)	489.79 ± 45.27	524.26 ± 55.34	5.283	< 0.001
CEA (ng/mL)	20.53 ± 3.02	21.33 ± 4.14	1.708	0.089
CA199 (U/mL)	39.58 ± 6.03	45.25 ± 8.59	5.906	< 0.001
AST (U/L)	53.12 ± 9.28	57.25 ± 11.34	3.109	0.002
ALT (U/L)	49.59 ± 8.51	52.25 ± 9.23	2.332	0.021
GGT (U/L)	270.02 ± 10.11	290.05 ± 18.48	10.364	< 0.001
ALP (U/L)	339.06 ± 17.64	369.55 ± 22.36	11.724	< 0.001
ChE (U/L)	5151.29 ± 682.25	4997.25 ± 574.38	1.894	0.059
ALB (g/L)	37.06 ± 5.29	35.95 ± 4.51	1.751	0.081
TBIL (μmol/L)	18.97 ± 3.26	20.08 ± 4.21	2.283	0.023
Maximum diameter of tumor (cm)	4.84 ± 0.62	5.11 ± 0.72	3.045	0.003
Histological differentiation (N,%)				
High differentiation	47 (37.60)	35 (29.91)	4.310	0.116
Differentiation	50 (40.00)	42 (35.90)		
Low differentiation	28 (22.40)	40 (34.19)		
Tumor margin (N,%)				
Smooth	68 (54.40)	54 (46.15)	1.644	0.200
Rough	57 (45.60)	63 (53.85)		
High enhancement in the arterial phase (N,%)				
No	47 (37.60)	40 (34.19)	0.306	0.580
Yes	78 (62.40)	77 (65.81)		
Rapid washout during the portal phase (N,%)				
No	52 (41.60)	32 (27.35)	5.415	0.020
Yes	73 (58.40)	85 (72.65)		

BMI, body mass index; BCLC, Barcelona Clinic Liver Cancer staging; AFP, alpha-fetoprotein; CEA, carcinoembryonic antigen; CA199, Carbohydrate Antigen 199; AST, aspartate aminotransferase; ALT, alanine aminotransferase; GGT, γ-glutamyl transpeptidase; ALP, alkaline phosphatase; ChE, cholinesterase; ALB, albumin; TBIL, total bilirubin. The Kolmogorov-Smirnov test was used to assess normality of distribution. Continuous variables that met the criteria for normal distribution were expressed as mean ± standard deviation. Comparisons between groups were performed using the independent samples *t*-test. Categorical variables were expressed as counts and percentages. Comparisons between groups were performed using the chi-square test. *P* < 0.05 was considered statistically significant.

### Early recurrence of hepatocellular carcinoma after TACE was associated with significantly reduced TTP and mTT, and increased PI

**FIGURE 1:**
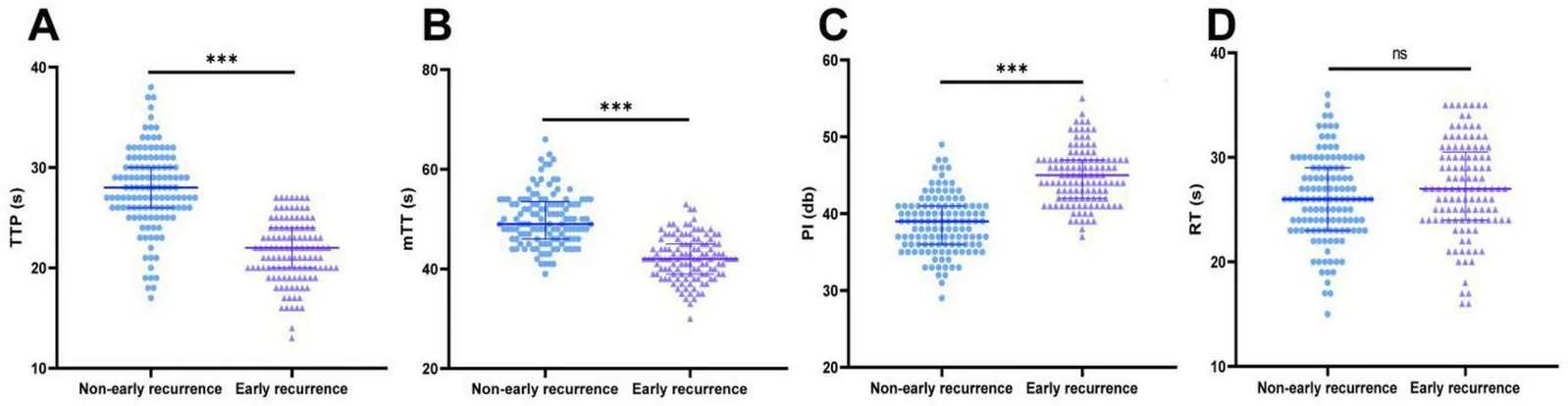
Comparison of CEUS parameters between the two groups. CEUS quantitative analysis was used to determine the time to peak (TTP) **(A)**, mean transit time (mTT) **(B)**, peak intensity (PI) **(C)**, and rise time (RT) **(D)**. The Kolmogorov-Smirnov test was used to assess normality of distribution. For non-normally distributed quantitative data in **(A–C)**, median (interquartile range) was used for presentation, and intergroup comparisons were performed using the Mann–Whitney U test. For normally distributed quantitative data in **(D)**, mean ± standard deviation was used for presentation, and between-group comparisons were performed using the independent samples *t*-test. ns indicates *P* > 0.05, ***P < 0.001.

We used CEUS quantitative analysis to assess TTP, mTT, PI, and RT. The results showed that TTP and mTT were significantly lower in the early recurrence group of hepatocellular carcinoma patients after TACE compared to the non-early recurrence group, while PI was significantly higher in the early recurrence group than in the non-early recurrence group (Figures 1A–C, all *P* < 0.001). Additionally, there was no statistically significant difference in RT between the two groups (Figure 1D, *P* > 0.05).

### Reduced TTP and mTT, and elevated PI are independent risk factors for early recurrence after TACE in hepatocellular carcinoma patients

To further analyze the risk factors influencing early recurrence after TACE in hepatocellular carcinoma patients, we used disease recurrence status (0 = no recurrence, 1 = recurrence) as the dependent variable. Subsequently, we analyzed the following variables from the clinical baseline data in Table 1: age, gender, BMI, smoking and drinking history, hepatitis B, BCLC staging, Child-Pugh classification, AFP, CEA, CA199, AST, ALT, GGT, ALP, ChE, ALB, TBIL, tumor maximum diameter, histological differentiation grade, CEUS examination findings, and CEUS parameters (TTP, mTT, PI) as independent variables in univariate logistic regression analysis. Subsequently, variables with *P* < 0.05 were included as independent variables in multivariable logistic regression analysis using a forward stepwise selection method (entry criterion *P* < 0.05, removal criterion *P* > 0.10). The results showed that elevated levels of AFP, CA199, GGT, and PI were independent risk factors for early recurrence after TACE in hepatocellular carcinoma patients, while prolonged TTP and mTT values were independent protective factors (all *P* < 0.05, Tables 2A,B). Odds ratios (ORs) with 95% confidence intervals (CIs) were reported instead of hazard ratios. >*H*2<TTP, mTT, and PI can assist in predicting early recurrence after TACE in hepatocellular carcinoma patients

**TABLE 2A:** Univariate logistic regression analysis of risk factors for early recurrence after TACE in patients with hepatocellular carcinoma.

Variable	*P*-value	OR	95% CI
Age (years)	0.969	1.001	0.968–1.034
BMI (kg/m^2^)	0.433	1.038	0.946–1.138
Gender (male = 1, female = 0)	0.935	0.984	0.668–1.449
Smoking history (yes = 1, no = 0)	0.540	1.128	0.768–1.656
Drinking history (yes = 1, no = 0)	0.581	1.111	0.764–1.615
Hepatitis B (yes = 1, no = 0)	0.606	1.135	0.701–1.837
BCLC staging (Stage C = 1, Stage B = 0)	0.593	1.123	0.734–1.717
Child-Pugh classification (Grade A = 1, Grade B = 0)	0.852	0.966	0.672–1.389
AFP (ng/mL)	< 0.001	1.010	1.006–1.013
CEA (ng/mL)	0.059	1.049	0.998–1.103
CA199 (U/mL)	< 0.001	1.060	1.038–1.081
AST (U/L)	0.001	1.030	1.011–1.049
ALT (U/L)	0.019	1.026	1.004–1.048
GGT (U/L)	< 0.001	1.047	1.037–1.057
ALP (U/L)	< 0.001	1.034	1.026–1.041
ChE (U/L)	0.086	1.000	0.999–1.000
ALB (g/L)	0.111	0.971	0.936–1.007
TBIL (μmol/L)	0.026	1.057	1.007–1.109
Maximum diameter of tumor (cm)	0.013	1.412	1.077–1.851
Histological differentiation (High vs. medium)	0.509	1.163	0.743–1.822
Histological differentiation (High vs. low)	0.072	1.516	0.963–2.387
Tumor margin (irregular = 1, smooth = 0)	0.337	1.195	0.831–1.719
High enhancement in arterial phase (yes = 1, no = 0)	0.594	1.110	0.757–1.626
Rapid washout of portal vein phase (yes = 1, no = 0)	0.031	1.565	1.042–2.351
TTP (s)	< 0.001	0.807	0.772–0.843
mTT (s)	< 0.001	0.848	0.818–0.878
PI (dB)	< 0.001	1.223	1.176–1.272

BMI, body mass index; BCLC, Barcelona Clinic Liver Cancer staging; AFP, alpha-fetoprotein; CEA, carcinoembryonic antigen; CA199, carbohydrate antigen 199; AST, aspartate aminotransferase; ALT, alanine aminotransferase; GGT, γ-glutamyl transpeptidase; ALP, alkaline phosphatase; ChE, cholinesterase; ALB, albumin; TBIL, total bilirubin; TTP, time to peak; mTT, mean transit time; PI, peak intensity; OR, odds ratio; CI, confidence interval.

**TABLE 2B:** Multivariable logistic regression analysis of independent risk factors for early recurrence after TACE in patients with hepatocellular carcinoma.

Variable	*P*-value	OR	95% CI
AFP (ng/mL)	0.046	1.003	1.000–1.007
CA199 (U/mL)	0.038	1.023	1.001–1.045
GGT (U/L)	0.029	1.013	1.001–1.025
TTP (s)	< 0.001	0.877	0.831–0.926
mTT (s)	< 0.001	0.906	0.869–0.945
PI (dB)	< 0.001	1.096	1.041–1.154

Only variables with *P* < 0.05 in multivariable analysis are presented. AFP, alpha-fetoprotein; CA199, carbohydrate antigen 199; GGT, γ-glutamyl transpeptidase; TTP, time to peak; mTT, mean transit time; PI, peak intensity; OR, odds ratio; CI, confidence interval. Elevated levels of AFP, CA199, GGT, and PI were independent risk factors for early recurrence, while prolonged TTP and mTT were independent protective factors. The final model demonstrated good fit according to the Hosmer–Lemeshow test (χ^2^ = 8.24, *P* = 0.409).

**FIGURE 2:**
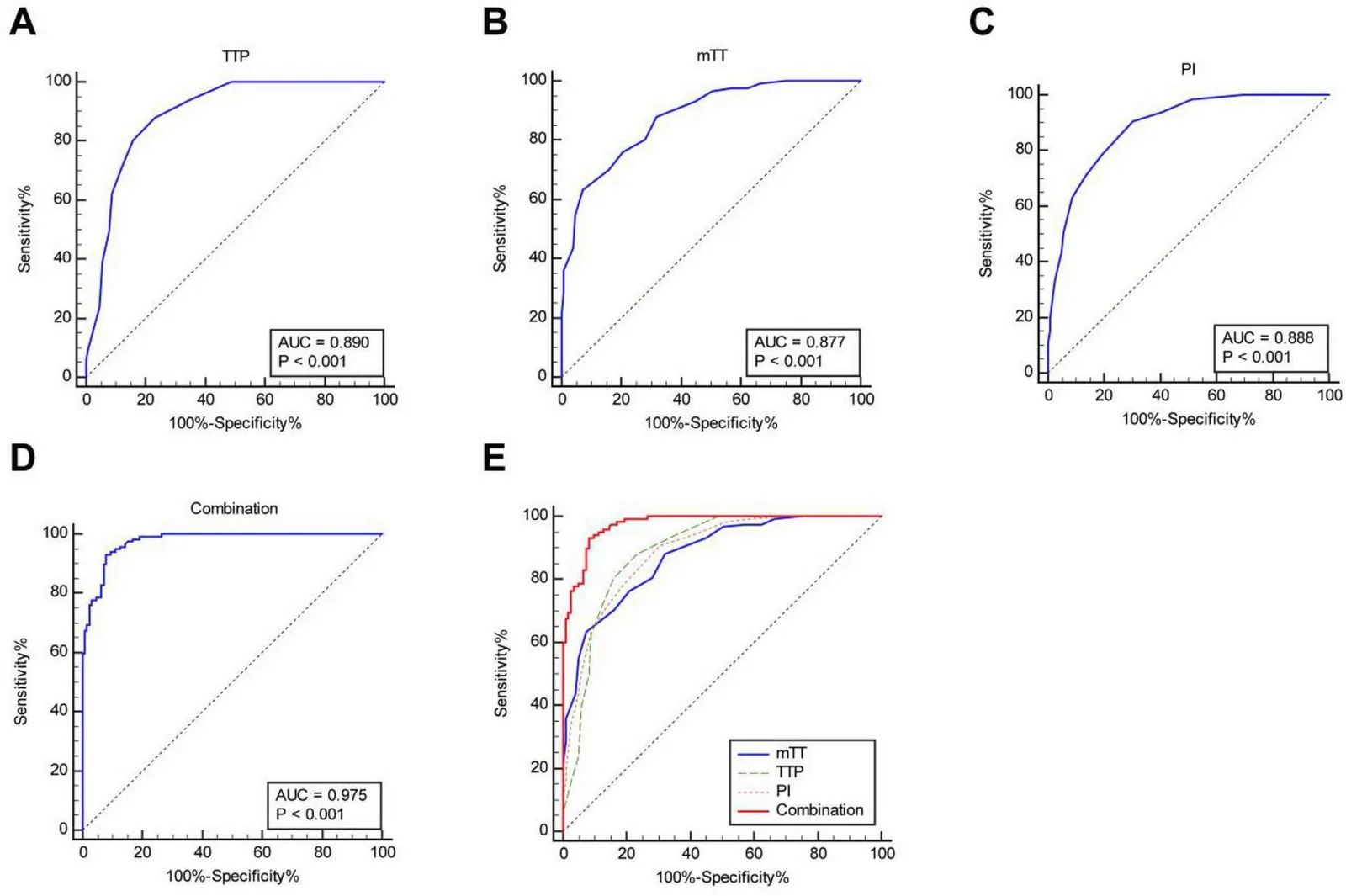
ROC curve analysis of TTP, mTT, and PI alone and in combination for predicting early recurrence after TACE in patients with hepatocellular carcinoma. ROC curve analysis of the predictive value of TTP **(A)** and mTT **(B)**, PI **(C)** alone and in combination **(D)** for early recurrence after TACE in patients with hepatocellular carcinoma. **(E)** Comparison of AUC among single indicators and combined model.

We plotted ROC curves to analyze the predictive value of TTP, mTT, and PI for early recurrence after TACE in hepatocellular carcinoma patients. The results showed that the AUC for TTP in predicting early recurrence after TACE in hepatocellular carcinoma patients was 0.890 (95% CI: 0.844–0.927), with a cutoff value of 25 s, a sensitivity of 88.03%, and a specificity of 76.80%; The AUC for mTT in predicting early recurrence after TACE in hepatocellular carcinoma patients was 0.877 (95% CI: 0.829–0.916), with a cutoff value of 43 s, sensitivity of 63.25%, and specificity of 92.80%; The AUC of PI for predicting early recurrence after TACE in liver cancer patients was 0.888 (95% CI: 0.841–0.925), with a cutoff value of 40 dB, a sensitivity of 90.60%, and a specificity of 69.60%. The AUC for the combined detection of the three factors was 0.975 (95% CI: 0.947–0.991), with a sensitivity of 93.16% and a specificity of 92.00% (Figure 2). To address potential overfitting concerns, internal validation using 1,000 bootstrap resamples was performed, yielding a bias-corrected AUC of 0.968 (95% CI: 0.941–0.985) for the combined model, indicating excellent robustness. The Hosmer–Lemeshow test for the combined model showed good calibration (χ^2^ = 6.32, *P* = 0.612), and the calibration plot demonstrated close agreement between predicted and observed probabilities of early recurrence. Further comparison analysis of the AUC using MedCalc software showed that the AUC for the combined prediction of early recurrence after TACE in liver cancer patients was significantly superior to that of TTP, mTT, and PI when tested individually (all *P* < 0.001, Table 3). The final multivariate Cox model specification is as follows: log[h(t)/h0(t)] = β_1_(AFP) + β_2_(CA199) + β_3_(GGT) + β_4_(PI) + β_5_(TTP) + β_6_(mTT), where all continuous variables were entered as continuous measures without dichotomization, and proportional hazards assumption was verified using Schoenfeld residuals (global test *P* = 0.213).

**TABLE 3:** Comparison of the predictive value of TTP, mTT, and PI alone and in combination for early recurrence after TACE in patients with hepatocellular carcinoma.

Indicator	AUC	95%CI	Sensitivity (%)	Specificity (%)
TTP	0.890	0.844–0.927	88.03	76.80
mTT	0.877	0.829–0.916	63.25	92.80
PI	0.888	0.841–0.925	90.60	69.60
Combination	0.975	0.947–0.991	93.16	92.00
TTP vs. combination	*P* < 0.001			
mTT vs. combination	*P* < 0.001			
PI vs. combination	*P* < 0.001			

Multiple ROC curve area under the curve (AUC) comparisons were performed using the Delong test in MEDCALC software (20.0.15, MedCalc Software Ltd., Ostend, Belgium). Internal validation of the combined model using 1,000 bootstrap resamples yielded a bias-corrected AUC of 0.968 (95% CI: 0.941–0.985). The Hosmer–Lemeshow test for the combined model showed good calibration (χ^2^ = 6.32, *P* = 0.612).

### Combined prediction model specification and cut-off value

The combined prediction model was constructed using logistic regression with backward stepwise selection. The final model formula for calculating the combined risk score (P) for early recurrence is as follows: Logit(P) = ln[P/(1-P)] = −8.432 + 0.003 × (AFP) + 0.023 × (CA199) + 0.013 × (GGT) + 0.096 × (PI) − 0.123 × (TTP) − 0.094 × (mTT) where AFP is in ng/mL, CA199 in U/mL, GGT in U/L, PI in dB, TTP in seconds, and mTT in seconds. The optimal cut-off value for the predicted probability (P) was determined using the Youden index (maximizing sensitivity + specificity − 1). The optimal cut-off value was 0.382, meaning that patients with a predicted probability ≥ 0.382 were classified as high-risk for early recurrence. At this cut-off, the sensitivity was 93.16%, specificity 92.00%, positive predictive value (PPV) 91.5%, and negative predictive value (NPV) 93.5%.

### Low TTP and mTT, and high PI increase the risk of early recurrence after TACE in hepatocellular carcinoma patients

**FIGURE 3:**
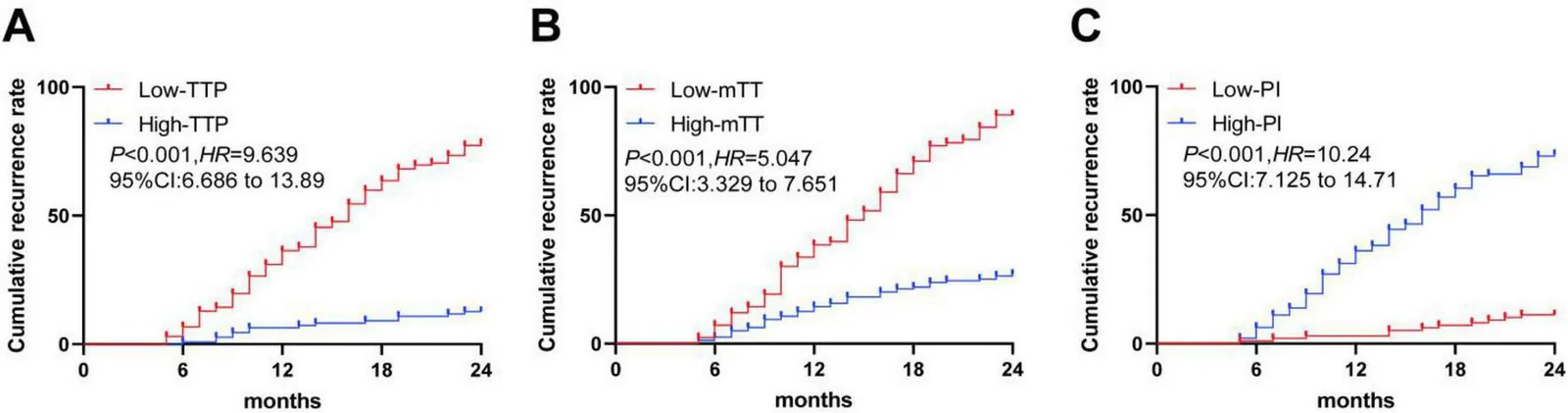
Low TTP and mTT, high PI increase the risk of early recurrence after TACE in patients with hepatocellular carcinoma. Kaplan–Meier curves showing recurrence-free survival (RFS) stratified by TTP **(A)**, mTT **(B)**, and PI **(C)**. The log-rank test was used for between-group comparisons. Number-at-risk tables are displayed below each curve. Low TTP (≤ 25 s) and low mTT (≤ 43 s) were associated with significantly shorter RFS, while high PI (> 40 dB) was associated with significantly shorter RFS (all *P* < 0.001).

Using the cutoff value (25 s) from the ROC curve analysis of TTP, hepatocellular carcinoma patients were divided into a low-TTP group (≤ 25, *N* = 132) and a high-TTP group (> 25, *N* = 110); Using the cutoff value from the ROC curve analysis of mTT (43 s) as the cutoff point, liver cancer patients were divided into a low-mTT group (≤ 43, *N* = 83) and a high-mTT group (> 43, *N* = 159); Using the cutoff value from the ROC curve analysis of PI (40 dB), liver cancer patients were divided into a low-PI group (≤ 40, *N* = 98) and a high-PI group (> 40, *N* = 144). Further comparison of early recurrence rates after TACE surgery among the groups revealed that the disease recurrence rates in the low-TTP group and low-mTT group were 78.03% and 89.16%, respectively, significantly higher than those in the high-TTP group (12.73%) and high-mTT group (27.04%) (all *P* < 0.001). The disease recurrence rate in the low-PI group (11.22%) was significantly lower than that in the high-PI group (73.61%) (*P* < 0.001) (Table 4). Subsequently, according to the results of the Kaplan–Meier curve, the KM curves of patients in the low-TTP and low-mTT groups shifted to the left compared with those in the high-TTP and high-mTT groups, while the KM curve of patients in the high-PI group shifted to the left compared with that in the low-PI group (*P* < 0.001), suggesting that low TTP and mTT, as well as high PI, increase the risk of early recurrence after TACE in hepatocellular carcinoma patients, as shown in Figure 3.

**TABLE 4:** Early recurrence of hepatocellular carcinoma after TACE in patients with different TTP, mTT, and PI values.

Indicator	No recurrence (*N*,%)	Recurrence (*N*,%)	χ^2^	*P*
Low-TTP group (*N* = 132)	29 (21.97)	103 (78.03)	102.460	< 0.001
High-TTP group (*N* = 110)	96 (87.27)	14 (12.73)		
Low-mTT group (*N* = 83)	9 (10.84)	74 (89.16)	84.247	< 0.001
High-mTT group (*N* = 159)	116 (72.96)	43 (27.04)		
Low-PI group (*N* = 98)	87 (88.78)	11 (11.22)	90.885	< 0.001
High-PI group (*N* = 144)	38 (26.39)	106 (73.61)		

## Discussion

Hepatocellular carcinoma often presents insidiously, with no symptoms or non-specific gastrointestinal symptoms such as abdominal distension and loss of appetite in the early stages, which are easily overlooked by patients. By the time patients notice significant discomfort, the disease has often progressed to the intermediate or advanced stages, rendering surgical treatment ineligible (14). Interventional therapy is one of the primary treatment modalities for liver cancer patients. Among these, TACE involves the infusion of chemotherapy drugs and embolization of tumor-supplying arteries to induce tumor apoptosis and control disease progression. However, the overall efficacy remains suboptimal, and patients face a high risk of recurrence post-treatment (15).

Currently, clinical assessments of treatment outcomes and recurrence in liver cancer patients are primarily based on tumor marker levels, with carcinoembryonic antigen (CEA) and alpha-fetoprotein (AFP) serving as key indicators. These markers are secreted by tumor cells, and elevated levels indicate tumor growth (16). However, studies have shown that CEA and AFP have limited specificity and may be overexpressed under inflammatory stimulation, limiting their clinical application (17). Imaging is an important tool for diagnosing liver cancer and assessing prognosis, enabling observation of tumor size, nature, and stage, thereby guiding clinical diagnosis and treatment.

Ultrasound, as a commonly used non-invasive imaging modality, offers radiation-free examinations and reproducible procedures. It is widely employed in clinical practice for the diagnosis of various tumors and can capture blood flow signals within and around tumors, demonstrating significant clinical value (18, 19). Reports indicate that CEUS can assess tumor angiogenesis within hepatocellular carcinoma following anti-angiogenic therapy, ablation, and transarterial chemoembolization (TACE) (20, 21). CEUS quantitative analysis utilizes time-intensity curves (TICs) to analyze ultrasound contrast images. Through quantitative analysis of TICs, multiple parameters such as PI, TTP, and mTT can be obtained. Early-stage tumors can secrete pro-angiogenic factors or inhibit anti-angiogenic factors, thereby inducing endothelial cell proliferation and migration, angiogenesis, and tubulogenesis, leading to changes in blood flow perfusion. CEUS can indirectly indicate blood flow perfusion by obtaining parameters through quantitative analysis, thereby determining the presence of tumor vessels and inferring tumor classification. In recent years, numerous studies have applied CEUS to evaluate the efficacy of tumor treatment (22, 23). CEUS can detect residual tumor lesions after TACE, with sensitivity and specificity reaching 94.7 and 80.0%, respectively (24). TTP, mTT, PI, and RT are ultrasound contrast parameters, where TTP and mTT reflect the time to reach peak contrast agent concentration and total transit time, respectively; PI indicates contrast intensity; and RT is an important parameter reflecting tissue microcirculatory perfusion velocity. The results of this study showed that TTP and mTT were significantly lower in early recurrence group patients with hepatocellular carcinoma after TACE compared to non-early recurrence group patients, while PI was higher in the early recurrence group. There was no statistically significant difference in RT between the two groups. This suggests that the shortening of TTP and mTT and the elevation of PI may reflect the unique vascular biological characteristics of recurrent hepatocellular carcinoma. The shortening of TTP and mTT and the increase in PI indicate that the contrast agent’s transmission speed in tumor tissue has accelerated, with peak time occurring earlier. This may be due to the rich new blood vessels in liver cancer tissue, incomplete vascular wall structure, and increased vascular permeability, leading to rapid contrast agent entry into tumor tissue and reaching peak concentration. Additionally, the formation of arteriovenous fistulas and collateral circulation in tumor tissue may also accelerate contrast agent transmission and distribution.

On the other hand, our study results indicate that elevated levels of alpha-fetoprotein (AFP), carbohydrate antigen 199 (CA19-9), gamma-glutamyl transpeptidase (GGT), and peak intensity (PI) are independent risk factors for early recurrence after transarterial chemoembolization (TACE) in hepatocellular carcinoma patients, while prolonged time to peak (TTP) and mean transit time (mTT) are independent protective factors. AFP is a specific tumor marker for hepatocellular carcinoma, and its elevated levels are typically closely associated with increased tumor burden and enhanced proliferative activity (25). Elevated AFP levels may reflect active tumor cell proliferation, indicating stronger invasiveness and metastatic potential, thereby increasing the risk of postoperative recurrence. AFP may enhance tumor malignancy by promoting tumor angiogenesis and altering the tumor microenvironment. Although CA19-9 is traditionally considered a biomarker for intrahepatic cholangiocarcinoma, accumulating evidence has demonstrated that CA19-9 can also be elevated in a subset of HCC patients, particularly those with aggressive tumor biology, vascular invasion, or poor differentiation (26). In HCC, elevated CA19-9 levels have been associated with increased tumor invasiveness, higher rates of extrahepatic metastasis, and worse prognosis. The significant elevation of CA19-9 in the early recurrence group of our study may reflect a more aggressive HCC phenotype rather than inadvertent inclusion of ICC or cHCC-CCA patients, especially given that all included patients met strict diagnostic criteria (pathological confirmation with Hep Par-1/glypican-3 positivity or LI-RADS LR-5 classification). CA199 is a broad-spectrum tumor marker, and its elevation in hepatocellular carcinoma may indicate strong tumor invasiveness or the presence of extrahepatic metastasis (26). GGT is one of the liver function indicators, and its elevation may reflect hepatocyte damage or active tumor cell proliferation. In liver cancer patients, elevated GGT may be associated with increased metabolic demands caused by tumor cell proliferation, as well as liver function abnormalities resulting from hepatocyte damage (27). GGT is involved in glutathione metabolism, and its elevation may lead to increased oxidative stress and cumulative DNA damage, thereby promoting gene mutations and malignant transformation in tumor cells, increasing the risk of postoperative recurrence. Prolonged TTP and mTT may reflect slowed blood flow velocity within tumor tissue, resulting in more uniform distribution of contrast agents within tumor tissue. This may be related to changes in tumor tissue structure or treatment response, such as increased blood flow resistance due to tumor tissue fibrosis or necrosis, leading to slowed blood flow velocity.

Additionally, the study results showed that the AUC values for TTP, mTT, and PI in predicting early recurrence after TACE in liver cancer patients were 0.890, 0.877, and 0.888, respectively. The AUC for combined testing of all three was 0.975, significantly superior to testing TTP, mTT, or PI individually. Low TTP and mTT, along with high PI, increased the risk of early recurrence after TACE in liver cancer patients. This suggests that the combined detection of TTP, mTT, and PI significantly enhances the predictive efficacy of early recurrence after TACE in hepatocellular carcinoma patients, providing a precise risk stratification tool for clinical practice. Its high sensitivity and specificity aid in optimizing individualized treatment strategies and improving patient outcomes. For patients predicted to have a high risk of recurrence, follow-up intervals can be shortened (e.g., every 1–2 months) and the frequency of imaging examinations (e.g., contrast-enhanced CT/MRI) increased to promptly detect recurrence and implement intervention measures. For patients with low recurrence risk, follow-up intervals can be appropriately extended, reducing unnecessary examinations and lowering medical costs. By precisely predicting recurrence risk, overtreatment of low-risk patients can be avoided, reducing unnecessary medical expenses. Additionally, early intervention for high-risk patients can lower the incidence of late recurrence (e.g., extrahepatic metastasis), further alleviating the economic burden on patients and society. It also provides evidence for new treatment targets and multimodal imaging fusion.

### Comparison with prior work

A recent study by Huang et al. (18) investigated the value of CEUS parameters in predicting early recurrence after curative resection of HCC. While their study focused on surgical patients, our study specifically addresses early recurrence after TACE, which represents a distinct patient population with unresectable or intermediate-stage HCC. Several key differences warrant mention: First, tumor hemodynamics may differ between resectable and unresectable HCC, potentially affecting CEUS parameter profiles. Second, TACE induces ischemic necrosis and inflammatory responses that may influence recurrence patterns differently from surgical resection. Third, our study incorporated a combined model integrating three CEUS parameters (TTP, mTT, PI) with serum biomarkers (AFP, CA199, GGT), whereas Huang et al. focused primarily on qualitative CEUS features. Despite these differences, both studies consistently identified shorter TTP and mTT as predictors of early recurrence, reinforcing the biological link between rapid contrast wash-in/washout and aggressive tumor behavior. Our study extends the literature by providing a quantitative, internally validated prediction model specifically for TACE-treated patients, addressing an important gap as most existing CEUS prediction models have focused on surgical cohorts.

In summary, the combined detection of TTP, mTT, and PI can assist in predicting early recurrence after TACE in patients with hepatocellular carcinoma. The exceptionally high AUC (0.975) observed in our study warrants discussion. Several factors may contribute to this strong discrimination: First, CEUS parameters (TTP, mTT, PI) directly quantify tumor hemodynamics, which are biologically closely linked to angiogenesis and tumor aggressiveness-key drivers of early recurrence. Second, the combined model integrated both CEUS parameters and serum biomarkers (AFP, CA199, GGT), capturing complementary dimensions of tumor biology. Third, our strict endpoint definition (imaging-confirmed recurrence within 24 months) and complete follow-up (no loss to follow-up for the primary outcome) minimized outcome misclassification bias. Importantly, internal validation using both 1,000 bootstrap resamples (bias-corrected AUC: 0.968, 95% CI: 0.941–0.985) and 10-fold cross-validation (mean cross-validated AUC: 0.959, 95% CI: 0.932–0.976) confirmed model robustness, suggesting that the observed performance is not merely due to overfitting. The optimism-corrected AUC after bootstrapping was 0.964, indicating minimal optimism (0.011) in the apparent AUC. Nevertheless, external validation in independent, preferably multi-center cohorts with larger sample sizes remains essential before clinical implementation, as internal validation techniques cannot fully account for between-center variability in patient populations, TACE protocols, and CEUS equipment. However, this study also has the following limitations: This was a single-center retrospective study with a relatively small sample size, which inevitably introduces potential bias. Although bootstrap internal validation suggested robustness, the lack of an external validation cohort remains a major limitation, and the reported AUC may still be optimistic compared to performance in real-world clinical settings. Larger sample sizes are needed in future studies to validate these results. Additionally, the prediction of early recurrence requires validation through long-term follow-up results; however, some studies may have insufficient follow-up periods or high loss-to-follow-up rates, leading to inadequate assessment of long-term recurrence risk. Our study had no loss to follow-up within the 24-month observation period, but longer-term outcomes (> 24 months) were not assessed. Furthermore, there are few studies on the combined application of ultrasound contrast parameters with other clinical indicators (such as AFP, imaging characteristics) or molecular markers, which may limit their clinical utility. Additionally, the generalizability of our findings may be constrained by the specific TACE protocol used (pirarubicin + iodized oil + gelatin sponge particles) and the patient population (predominantly hepatitis B-related HCC from a single center in China). Further studies are needed to extend follow-up periods, explore the predictive efficacy of ultrasound contrast parameters for long-term liver cancer recurrence, and integrate them with existing predictive tools, with the aim of providing new insights for the clinical diagnosis and treatment of liver cancer.

## Data Availability

The original contributions presented in this study are included in this article/supplementary material, further inquiries can be directed to the corresponding author.
